# Correlation of foot process effacement with clinicopathological features and short-term prognosis in IgA nephropathy

**DOI:** 10.1080/07853890.2026.2674376

**Published:** 2026-05-20

**Authors:** Yu Wang, Yang Yang, Xiaoli Wen, Lu Yi, Fang Zeng, Baoqin Zhou, Lijuan Wang, Wenjun Yan, Daijin Ren, Shizhang Xu, Yebei Li, Dehui Liu, Kaiping Luo, Hong Jiang, Gaosi Xu

**Affiliations:** ^a^ Department of Nephrology, The Second Affiliated Hospital, Jiangxi Medical College, Nanchang University, Nanchang, China; ^b^ Department of Nephrology, The Affiliated Ganzhou Hospital, Jiangxi Medical College, Nanchang University, Ganzhou, P. R. China; ^c^ Department of Nephrology, Xinyu People’s Hospital, Xinyu, China; ^d^ Department of Nephrology, Shangrao People’s Hospital, Shangrao, China; ^e^ Department of Nephrology, The First Affiliated Hospital of Gannan Medical University, Ganzhou, PR China; ^f^ Department of Health Management Center, Jiangxi Provincial People’s Hospital, Nanchang, PR China; ^g^ Department of Nephrology, Yichun People’s Hospital, Yichun, China; ^h^ Department of Health Management Center, the First Affiliated Hospital, Jiangxi Medical College, Nanchang University, Nanchang, China; ^i^ Kidney Disease Center, The First Affiliated Hospital, College of Medicine, Zhejiang University, Hangzhou, Zhejiang Province, PR China

**Keywords:** IgA nephropathy, podocyte, foot process effacement

## Abstract

**Objective:**

To compare clinicopathological characteristics and short-term renal outcomes of IgA nephropathy (IgAN) patients with varying degrees of foot process effacement (FPE).

**Methods:**

This multicenter retrospective study evaluated 1,001 biopsy-proven IgAN patients grouped by FPE severity (mild, moderate, severe). Regression models identified factors associated with different FPE degrees. Short-term outcomes in a 1-year follow-up subset were analyzed using overlap weighting, Cox regression, and Kaplan-Meier analysis.

**Results:**

The cohort comprised 38.8% mild, 49.6% moderate, and 11.6% severe FPE cases. Greater FPE severity corresponded with elevated blood pressure, proteinuria, and anemia, alongside decreased estimated glomerular filtration rate (eGFR) and albumin levels. Pathologically, worsening FPE correlated with advanced mesangial hyperplasia and tubulointerstitial damage. Severe FPE was distinctively associated with higher serum phosphorus, endocapillary hypercellularity (E1), and progressive interstitial fibrosis (T). Among 486 followed patients, complete remission rates were significantly lower in the severe FPE group than in the mild group (HR = 0.475, *p* = 0.046).

**Conclusions:**

The degree of FPE in IgAN was significantly positively correlated with proteinuria, hypoalbuminemia, eGFR decline, mesangial hypercellularity, and tubular atrophy/interstitial fibrosis. Severe FPE is an independent risk factor influencing short-term renal outcomes in IgAN patients.

## 1. Introduction

Immunoglobulin A nephropathy (IgAN) is the most common pattern of primary glomerular disease worldwide [1,2]. Characterized by mesangial deposition of galactose-deficient IgA1 (Gd-IgA1) immune complexes, IgAN progresses to end-stage kidney disease (ESKD) in 20–40% of patients within two decades, despite optimized supportive care [3,4].

In recent years, the pathogenesis of IgAN have been made significant breakthroughs. While the multi-hit hypothesis remains a cornerstone, recent multiomics and single-cell studies have revealed a much more intricate interplay of immune and renal cells in IgAN pathogenesis. Expanded populations of mucosal-associated invariant T cells and T helper 17 (Th17) cells promote the differentiation of B cells into IgA-secreting plasmablasts *via* IL-17 signaling pathways [5–7]. Within the kidney, Gd-IgA1 immune complexes activate mesangial cells, upregulating Toll-like receptor 4 (TLR4) and NLRP3 inflammasomes, and activating MAPK/ERK and NF-κB signaling pathways, leading to the release of proinflammatory cytokines such as IL-6 and tumor necrosis factor-α (TNF-α) [8,9]. Glomerular immune complexes containing Gd-IgA1 activate the alternative complement pathway (AP) by using properdin to stabilize C3 convertase formation. This activation not only amplifies mesangial injury through C5a-driven inflammation and MAC-induced cell lysis, but also drives progressive tubulointerstitial fibrosis by attracting macrophages and activating endothelial cells [10,11].

Podocytes are highly differentiated epithelial-like cells that, together with endothelial cells and the glomerular basement membrane, form the major components of the glomerular filtration barrier, which is essential for maintaining normal kidney function. Podocyte injury is a common pathological feature in various glomerular diseases, including IgAN, minimal change disease, focal segmental glomerulosclerosis, diabetic nephropathy, membranous nephropathy, and lupus nephritis [12]. The strong association between podocyte injury and the severity of IgAN is now widely recognized [13].

The Oxford classification MEST-C scores, proposed by an international working group in 2009 and updated in 2016, is the most widely accepted histological classification system in IgAN [14,15]. The International IgAN Prediction Tool, endorsed by the Kidney Disease Improving Global Outcomes (KDIGO) Guidelines, incorporates Oxford Classification (MEST-C) scores from the incident kidney biopsy as a significant component of the risk calculator that has been extensively applied in clinical practice [16]. Notably, Bellur et al. identified a pathological subtype of segmental glomerulosclerosis, strongly associated with podocyte injury, characterized by an active disease pattern detectable only by renal biopsy [17]. Meanwhile, existing studies have demonstrated that foot process effacement (FPE) is an independent risk factor for adverse renal outcomes in patients with IgAN [18], underscoring the clinical importance of evaluating podocyte injury in disease management.

However, the relationships between FPE and the clinical features, pathological classification, and prognosis of IgAN have not been systematically investigated, and current KDIGO guidelines provide limited evidence for this patient subgroup. Therefore, we undertook this study to examine the associations between different degrees of FPE and clinicopathologic characteristics, as well as the short-term prognosis of patients with IgAN.

## 2. Methods

### 2.1. Ethics approval and consent to participate

This study was conducted in accordance with the Declaration of Helsinki and was approved by the Ethics Committee of the Second Affiliated Hospital of Nanchang University (Approval No. IIT-O-2025-195). The requirement for informed consent was waived due to the retrospective nature of the study.

### 2.2. Study design and population

This multicenter retrospective cohort study included patients diagnosed with IgAN by renal biopsy between January 2018 and July 2024. Patients were excluded if they were younger than 18 years of age, had secondary IgAN related to liver disease, lupus, or Henoch–Schönlein purpura, or had IgAN combined with ANCA-associated necrotizing glomerulonephritis, minimal change disease like IgAN, diabetic kidney disease, or hypertensive nephrosclerosis. There were no restrictions on baseline estimated glomerular filtration rate (eGFR) or proteinuria level; however, patients who were dialysis-dependent at baseline were excluded. A total of 1001 patients met the inclusion criteria and were enrolled in the study, of whom 486 had at least one year of follow-up data.

Baseline and follow-up clinical data included demographic characteristics, eGFR, hematuria, 24-h proteinuria, serum albumin (Alb), and pathological findings, including Oxford and Lee’s classifications. All baseline data were collected at the time of renal biopsy.

### 2.3. Pathology assessment

Renal biopsy specimens from all enrolled cases underwent a centralized re-evaluation using both light and electron microscopy. Histopathological lesions of IgAN were scored according to the internationally recognized MEST-C scoring system. Mesangial hypercellularity was scored as M1 when ≥50% of glomeruli contained at least one mesangial area with ≥4 mesangial cells. Endocapillary hypercellularity (E) was scored present (E1) or absent (E0). Based on the presence or absence of any segmental sclerosis or adhesion, segmental sclerosis (S) was scored as absent (S0) or present (S1). Tubular atrophy and interstitial fibrosis (T) were graded as T0 (≤25%), T1 (26%–50%), or T2 (>50%). Crescentic (C) was scored as C0 (0%), C1 (1%–24%), or C2 (≥25%) according to the proportion of glomeruli exhibiting cellular and/or fibro cellular crescents. Additionally, podocyte FPE was defined as the loss of the regular interdigitating pattern between adjacent podocyte foot processes [19]. The FPE burden was defined as the percentage of capillary loops exhibiting effacement relative to the total number of loops evaluated, with patients subsequently stratified into three categories: mild (<30%), moderate (30%–69%), and severe (≥70%). To ensure diagnostic precision, all histopathological parameters were independently reviewed by two renal pathologists. The final FPE ratio was determined as the mean of the two observers’ measurements, and any interpretational discrepancies were resolved through inter-observer consultation to reach a consensus.

### 2.4. Treatment regimens

Follow-up data were collected at 1, 3, 6, 9, and 12 months after diagnosis. Ultimately, 486 patients who met the criteria were included in the prognostic analysis. According to the treatment regimen, they were further stratified into those receiving glucocorticoids (GCs) and/or immunosuppressive therapy (IST) and those receiving supportive care alone. GCs therapy was defined as an initial dose of prednisone at 0.4-0.6 mg/kg per day, with a maximum dose of 60 mg/day, followed by a gradual taper and discontinuation within six months. IST included cyclophosphamide, cyclosporine, azathioprine, or mycophenolate mofetil, regardless of dosage or treatment duration. Supportive therapy consisted of treatment with angiotensin-converting enzyme inhibitors, angiotensin receptor blockers, sodium-glucose cotransporter inhibitor, or finerenone.

### 2.5. Outcome definitions

Complete remission (CR) was defined as proteinuria <0.3 g/24 h and eGFR ≥50 ml/min/1.73 m^2^, while partial remission (PR) was defined as a reduction of more than 50% from baseline proteinuria with an absolute level <1.0 g/24 h. The overall remission rate was calculated as the sum of CR and PR.

### 2.6. Statistical analysis

Missing data were handled using multiple imputation with 25 iterations. The normality of continuous variables was assessed by the Shapiro-Wilk test. Normally distributed data were expressed as mean ± standard deviation, and non-normally distributed data were expressed as medians and interquartile range (IQR). Comparisons of continuous variables among groups were performed using the Kruskal-Wallis test, while categorical variables were presented as counts and percentages and compared using the chi-square test. Correlations between FPE severity and clinical or pathological characteristics were evaluated using Spearman analysis. To further identify factors independently associated with FPE, multinomial logistic regression was performed using the mild group as the reference. Candidate variables were initially evaluated using univariate multinomial logistic regression and the likelihood ratio test (LRT, *p* < 0.05). Variance inflation factors (VIF) or generalized VIFs were computed to assess multicollinearity, and any variable with a VIF exceeding 5 was removed to ensure model stability. The remaining non-collinear factors were then entered into a multivariable multinomial logistic regression analysis. The final model was derived *via* backward stepwise selection. Effect sizes are reported as unadjusted and adjusted odds ratios (ORs and aOR) alongside their 95% confidence intervals (CIs). To explore the potential roles of eGFR and the platelet-to-albumin ratio (PAR) in the association between FPE severity and hemoglobin levels, we performed a mediation analysis. Indirect effects and their 95% CIs were estimated using a bootstrap method with 5,000 resamples.

For the prognostic analysis, only patients with follow-up of more than 1 year were included. To mitigate imbalance in baseline characteristics, we chose propensity score weighting. The propensity score was estimated using a logistic regression model. We included established risk factors and clinically relevant variables in our propensity score model, including age, systolic blood pressure (SBP), 24-h proteinuria, and eGFR, etc. We utilized overlap weighting as our primary weighting approach. The effectiveness of the weighting was assessed by examining the standardized mean differences (SMDs) of covariates between the two groups after weighting. SMDs less than 0.2 indicated adequate balance. To compare overall remission and CR across the three groups, weighted Cox regression models were employed. We calculated three models, of which Model 1 was adjusted for age, sex, body mass index (BMI), eGFR, and baseline proteinuria. Then, Model 2 further incorporated the MEST-C scores. Finally, Model 3 additionally accounted for treatment regimens. Regression coefficients, 95% CIs, and *P*-values were reported. A two-sided alpha level of 0.05 was used to determine statistical significance. All statistical analyses were performed using R 4.5.1.

## 3. Results

### 3.1. Comparison of clinicopathological characteristics among groups based on FPE

**Figure 1.**
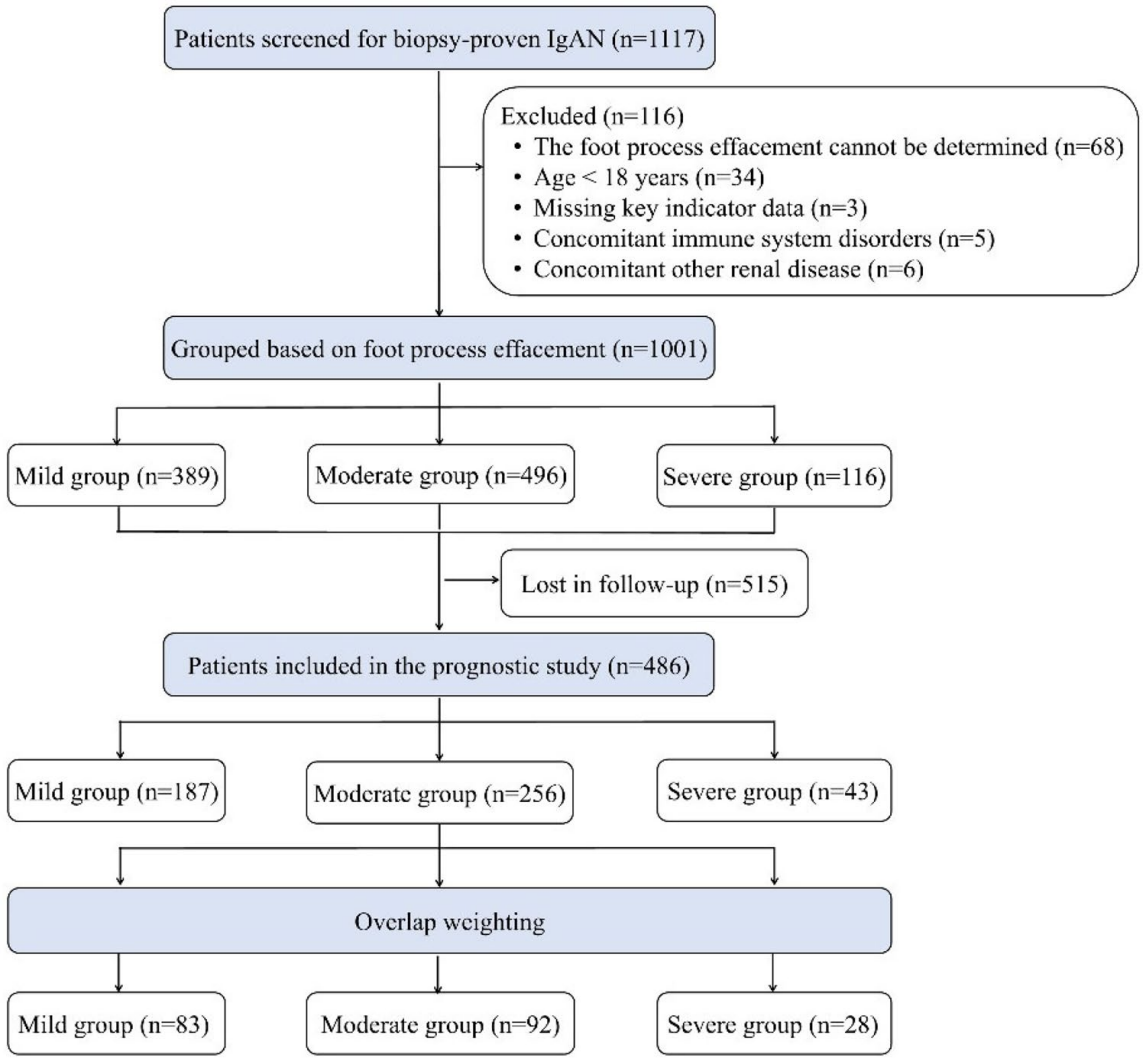
Flow diagram of the study population.

A total of 1001 patients with IgAN were included in this study, comprising 389 (38.8%) with mild, 496 (49.6%) with moderate, and 116 (11.6%) with severe FPE (Figure 1). There were no significant differences among the three groups in the prevalence of hypertension, diabetes, or hepatitis B infection. With increasing FPE severity, both systolic SBP and diastolic blood pressure (DBP) increased significantly (*p* = 0.003 and 0.043, respectively). The 24-h proteinuria level rose markedly from 1.5 g/24h in the moderate group to 3.7 g/24h in the severe group (*p* < 0.001), and the proportion of patients with nephrotic-range proteinuria (≥3.5 g/24h) reached 53.4% in the severe group, compared with 8.2% in the mild group. Serum total protein and Alb levels decreased progressively with worsening FPE (both *p* < 0.001), with hypoalbuminemia most pronounced in the severe group. Red blood cell count and hemoglobin levels also declined with increasing FPE severity (both *p* < 0.001); anemia was more prevalent in the severe group (hemoglobin ≥120 g/L: 54.3% vs 76.9%). Neutrophil counts and PAR increased with disease progression (*p* = 0.032 and <0.001, respectively), while lymphocyte counts decreased (*p* = 0.001).

Regarding renal function, both blood urea nitrogen and serum creatinine levels were significantly elevated in the severe group (*p* < 0.001), whereas eGFR declined markedly (*p* < 0.001); 59.5% of patients in the severe group had eGFR <60 ml/min/1.73 m^2^ (Table 1).

**Table 1.** Clinical characteristics of patients with IgAN with different degrees of foot process effacement.

Characters	Mild Group: < 30% (*n* = 389)	Moderate group: 30–69% (*n* = 496)	Severe group: ≥ 70% (*n* = 116)	*p-*value
Female (*n*)	234.0 (60.2)	295.0 (59.5)	61.0 (52.6)	0.328
Age (years)	38.0 [29.0, 47.0]	39.0 [29.0, 49.0]	41.5 [30.8, 54.0]	0.042
BMI (kg/m^2^)	23.5 [21.3, 26.3]	23.5 [21.6, 26.0]	23.8 [21.7, 26.5]	0.748
SBP (mmHg)	128.0 [116.0, 140.0]	129.0 [118.0, 142.0]	135.5 [120.8, 151.2]	0.003
DBP (mmHg)	84.0 [75.0, 93.0]	84.0 [77.0, 93.0]	88.0 [78.0, 99.0]	0.043
Hypertension (n)	105.0 (27.0)	136.0 (27.4)	38.0 (32.8)	0.454
Diabetes (n)	12.0 (3.1)	21.0 (4.2)	6.0 (5.2)	0.512
HBV (n)	62.0 (15.9)	84.0 (16.9)	24.0 (20.7)	0.489
24-hour proteinuria (g/24h)	1.1 [0.6, 2.0]	1.5 [0.8, 2.8]	3.7 [1.9, 5.4]	<0.001
<1	167.0 (42.9)	162.0 (32.7)	14.0 (12.1)	<0.001
1 to <3.5	190.0 (48.8)	241.0 (48.6)	40.0 (34.5)	
≥3.5	32.0 (8.2)	93.0 (18.8)	62.0 (53.4)	
Hematuria (*n*)	355.0 (91.3)	460.0 (92.7)	110.0 (94.8)	0.411
WBC (×10^9^/L)	6.6 [5.4, 7.9]	6.7 [5.6, 8.0]	7.1 [5.7, 8.7]	0.152
RBC (×10^12^/L)	4.5 [4.2, 5.0]	4.4 [4.0, 4.9]	4.2 [3.7, 4.7]	<0.001
Hb (g/L)	132.0 [120.0, 144.0]	129.0 [115.0, 143.0]	121.0 [107.0, 136.0]	<0.001
60 to <90	8.0 (2.1%)	18.0 (3.6%)	13.0 (11.2%)	<0.001
90 to<120	82.0 (21.1%)	139.0 (28.0%)	40.0 (34.5%)	
≥120	299.0 (76.9%)	339.0 (68.3%)	63.0 (54.3%)	
Plt (×10^9^/L)	257.0 [218.0, 302.0]	255.5 [216.0, 301.5]	264.5 [217.2, 310.8]	0.661
NE (×10^9^/L)	4.1 [3.2, 5.2]	4.2 [3.4, 5.4]	4.6 [3.4, 6.1]	0.032
LY (×10^9^/L)	1.8 [1.5, 2.2]	1.7 [1.4, 2.1]	1.6 [1.3, 2.1]	0.001
MO (×10^9^/L)	0.4 [0.3, 0.5]	0.4 [0.3, 0.5]	0.4 [0.3, 0.6]	0.245
Scr (μmol/L)	84.6 [67.5, 116.0]	99.9 [74.9, 132.2]	125.5 [89.4, 216.6]	<0.001
BUN (mmol/L)	5.2 [4.3, 6.7]	5.8 [4.4, 7.4]	7.6 [5.1, 10.3]	<0.001
eGFR (ml/min/1.73m^2^)	85.4 [59.5, 105.5]	69.3 [49.1, 94.0]	50.1 [28.4, 76.2]	<0.001
<60	98.0 (25.2)	191 (38.5)	69.0 (59.5)	<0.001
60 to <90	117.0 (30.1)	157.0 (31.7)	29.0 (25.0)	
≥90	174.0 (44.7)	148.0 (29.8)	18.0 (15.5)	
UA (umol/L)	365.0 [301.0, 455.0]	394.0 [325.8, 463.9]	411.3 [336.4, 479.8]	0.001
TP (g/L)	69.0 [64.7, 73.1]	68.7 [62.9, 73.1]	59.5 [51.3, 67.4]	<0.001
Alb (g/L)	41.3 [38.1, 43.8]	40.2 [36.0, 43.1]	32.5 [26.3, 39.4]	<0.001
ALT (U/L)	17.0 [12.2, 23.9]	16.4 [12.0, 25.7]	15.7 [11.0, 22.0]	0.185
AST (U/L)	20.0 [16.0, 24.9]	20.8 [17.4, 25.0]	19.0 [15.5, 23.0]	0.038
PAR*	6.2 [5.2, 7.5]	6.5 [5.3, 7.9]	7.8 [6.4, 10.8]	<0.001
TC (mmol/L)	4.9 [4.3, 5.5]	5.0 [4.3, 5.8]	5.4 [4.4, 6.2]	0.006
TG (mmol/L)	1.6 [1.0, 2.4]	1.7 [1.2, 2.5]	2.0 [1.3, 2.7]	0.002
Serum HDL (mmol/L)	1.3 [1.0, 1.5]	1.2 [1.0, 1.5]	1.2 [1.0, 1.5]	0.492
Serum LDL (mmol/L)	3.0 [2.5, 3.6]	3.0 [2.5, 3.6]	3.3 [2.8, 4.2]	0.001
Glu (mmol/L)	5.0 [4.6, 5.6]	5.2 [4.7, 5.7]	5.1 [4.6, 5.7]	0.111
Serum potassium (mmol/L)	4.0 [3.8, 4.2]	4.0 [3.8, 4.3]	4.1 [3.8, 4.5]	0.012
Serum calcium (mmol/L)	2.3 [2.2, 2.4]	2.3 [2.2, 2.4]	2.2 [2.0, 2.3]	<0.001
Phosphorus (mmol/L)	1.1 [1.0, 1.2]	1.1 [1.0, 1.2]	1.2 [1.1, 1.4]	<0.001
IgG (g/L)	11.8 [10.2, 13.3]	11.4 [9.7, 13.2]	9.2 [7.5, 12.1]	<0.001
lgA (g/L)	3.3 [2.6, 3.9]	3.3 [2.7, 3.9]	3.0 [2.3, 3.9]	0.030
IgM (g/L)	1.2 [0.9, 1.5]	1.2 [0.9, 1.5]	1.1 [0.9, 1.4]	0.179
C3 (g/L)	1.0 [0.9, 1.1]	1.0 [0.9, 1.1]	1.0 [0.9, 1.2]	0.001
C4 (g/L)	0.2 [0.2, 0.3]	0.3 [0.2, 0.3]	0.3 [0.2, 0.3]	0.010

Abbreviations: IgAN, Immunoglobulin A nephropathy; BMI, Body mass index; SBP, Systolic blood pressure; DBP, Diastolic blood pressure; WBC, White blood cell; RBC, Red blood cell; Hb, Hemoglobin; Plt, Platelet; NE, Neutrophil count; MO, Monocyte count; LY, Lymphocyte count; BUN, Blood urea nitrogen; Scr, Serum creatinine; eGFR, Estimated glomerular filtration rate; UA, Serum uric acid; TP, Total protein; Alb, Serum albumin; ALT, Alanine aminotransferase; AST, Aspartate aminotransferase; TC, Total cholesterol; TG, Triglycerides; HDL, High-density lipoprotein; LDL, Low-density lipoprotein; Glu, Glucose; K⁺, Potassium; Ca²⁺, Calcium; P, Phosphorus; IgG, Immunoglobulin G; IgA, Immunoglobulin A; IgM, Immunoglobulin M; C3, Complement 3; C4, Complement 4; HBV, Hepatitis B virus.

* PAR = Plt/Alb.

Based on the Oxford MEST-C scores, the proportions of M1 and E1 lesions were significantly higher in the severe group compared with the mild group. Tubulointerstitial lesions worsened with increasing FPE severity, with T2 and C2 lesions being significantly more common in the severe group (all *p* < 0.001). The degree of interstitial inflammation correlated positively with FPE (*p* < 0.001), with severe inflammation observed in 56.0% of patients in the severe group compared with 27.5% in the mild group. With increasing FPE, the proportion of intense C3 immunofluorescence decreased, whereas weak C3 deposition became more prevalent (Table 2).

**Table 2.** Pathological characteristics of patients with IgAN with different degrees of foot process effacement.

Characters	Mild group: < 30% (*n* = 389)	Moderate group: 30%-69% (*n* = 496)	Severe group: ≥ 70% (*n* = 116)	*p-*value
Oxford histologic score, *n* %				
M0	33 (8.5)	19 (3.8)	2 (1.7)	0.002
M1	356 (91.5)	477 (96.2)	114 (98.3)	
E0	264 (67.9)	290 (58.5)	45 (38.8)	< 0.001
E1	125 (32.1)	206 (41.5)	71 (61.2)	
S0	162 (41.6)	162 (32.7)	41 (35.3)	0.073
S1	227 (58.4)	333 (67.1)	75 (64.7)	
S2	0 (0.0)	1 (0.2)	0 (0.0)	
T0	331 (85.1)	388 (78.2)	65 (56.0)	< 0.001
T1	46 (11.8)	84 (16.9)	24 (20.7)	
T2	12 (3.1)	24 (4.8)	27 (23.3)	
C0	222 (57.1)	258 (52.0)	50 (43.1)	< 0.001
C1	154 (39.6)	219 (44.2)	51 (44.0)	
C2	13 (3.3)	19 (3.8)	15 (12.9)	
Lee’s classification, *n* %				
I	3 (0.8)	0 (0.0)	1 (0.9)	< 0.001
II	78 (20.1)	30 (6.0)	9 (7.8)	
III	173 (44.5)	285 (57.5)	31 (26.7)	
IV	90 (23.1)	123 (24.8)	25 (21.6)	
V	45 (11.6)	58 (11.7)	50 (43.1)	
Interstitial inflammation, *n* %				
Mild	148 (38.0)	141 (28.4)	21 (18.1)	< 0.001
Moderate	134 (34.4)	202 (40.7)	30 (25.9)	
Severe	107 (27.5)	153 (30.8)	65 (56.0)	
C3 Immunofluorescence, *n* %				
Negative	49 (12.6)	68 (13.7)	17 (14.7)	0.039
Weakly positive	80 (20.6)	138 (27.8)	36 (31.0)	
Strongly positive	260 (66.8)	290 (58.5)	63 (54.3)	
IgG Immunofluorescence, *n* %				
Negative	339 (87.1)	453 (91.3)	104 (89.7)	0.131
Positive	50 (12.9)	43 (8.7)	12 (10.3)	
IgA Immunofluorescence, n %				
Weakly positive	76 (19.5)	126 (25.4)	25 (21.6)	0.112
Strongly positive	313 (80.5)	370 (74.6)	91 (78.4)	

Abbreviations: Oxford classification: M, Mesangial hypercellularity; E, Endocapillary hypercellularity; S, Segmental glomerulosclerosis; T, Tubular atrophy/interstitial fibrosis; C, Crescents.

### 3.2. Multinomial logistic regression and spearman correlation analysis

To identify independent indicators of FPE severity, a backward stepwise multinomial logistic regression was performed using the mild group as the reference. The analysis demonstrated distinct clinical and pathological factors associated with moderate versus severe FPE (Table 3 and Supplement Table 1).

**Table 3.** Multivariate multinomial logistic regression analysis of clinical and pathological indicators associated with foot process effacement.

	Moderate group		Severe group	
Variables	aOR (95% CI)	*p*-value	aOR (95% CI)	*p*-value
24-h proteinuria (g/24h)	1.128 (1.018–1.250)	0.022	1.074 (0.952–1.211)	0.245
eGFR (ml/min/1.73m^2^)	0.992 (0.986–0.997)	0.004	0.991 (0.981–1.001)	0.077
Alb (g/L)	0.947 (0.910–0.986)	0.008	0.858 (0.811–0.909)	<0.001
ALT (U/L)	1.008 (1.000–1.017)	0.064	0.991 (0.972–1.010)	0.368
Serum calcium (mmol/L)	9.469 (2.380–37.673)	0.001	0.954 (0.123–7.387)	0.964
Phosphorus (mmol/L)	1.086 (0.525–2.249)	0.823	2.624 (1.029–6.688)	0.043
C3 (g/L)	0.136 (0.058–0.318)	<0.001	1.336 (0.506–3.525)	0.559
C4 (g/L)	10.429 (1.095–99.281)	0.041	1.328 (0.035–50.895)	0.879
Endocapillary hypercellularity (E)				
E0	Reference		Reference	
E1	1.206 (0.873–1.665)	0.255	2.111 (1.234–3.613)	0.006
Tubular atrophy/interstitial fibrosis (T)				
T0	Reference		Reference	
T1	2.034 (1.245–3.323)	0.005	2.383 (1.134–5.011)	0.022
T2	1.970 (0.847–4.581)	0.116	4.226 (1.604–11.137)	0.004
Lee’s classification				
I–II	Reference		Reference	
III	4.238 (2.556–7.026)	<0.001	1.700 (0.626–4.619)	0.298
IV	1.854 (1.003–3.428)	0.049	0.897 (0.291–2.766)	0.850
V	1.136 (0.533–2.420)	0.741	1.468 (0.446–4.837)	0.528
C3 Immunofluorescence				
Negative	Reference		Reference	
Weakly positive	1.214 (0.733–2.012)	0.451	1.704 (0.739–3.928)	0.211
Strongly positive	0.653 (0.414–1.029)	0.066	0.501 (0.231–1.084)	0.079

Note: The reference group for the dependent variable (foot process effacement) is the mild group. aOR, adjusted odds ratio; CI, confidence interval. The final multivariable model was constructed using backward stepwise selection based on the Akaike information criterion (AIC).

In the moderate FPE group, multiple clinical and pathological parameters demonstrated significant independent associations. Clinically, elevated levels of 24-h proteinuria (OR = 1.128, 95% CI: 1.018-1.250, *p* = 0.022), serum calcium (OR = 9.469, 95% CI: 2.380-37.673, *p* = 0.001), and complement C4 (OR = 10.429, 95% CI: 1.095-99.281, *p* = 0.041) were positively correlated with moderate FPE. Conversely, higher Alb (*p* = 0.008), eGFR (*p* = 0.004), and complement C3 (*p* < 0.001) were inversely associated with this severity level. Regarding histological characteristics, compared to patients with T0 lesions or mild classifications (Lee’s I-II), those with T1 lesions (*p* = 0.005) or Lee’s grades III (*p* < 0.001) and IV (*p* = 0.049) presented with higher odds of moderate FPE.

When analyzing the severe FPE group, the significance of several clinical indicators shifted, while the associations with histological lesions became more prominent. Among clinical parameters, decreased Alb remained significantly associated with severe FPE (OR = 0.858, 95% CI: 0.811-0.909, *p* < 0.001), and an elevated serum phosphorus level emerged as a unique correlate for this group (OR = 2.624, 95% CI: 1.029-6.688, *p* = 0.043). Pathologically, E1 exhibited a significant positive association (*p* = 0.006). Notably, the severity of T demonstrated a clear positive trend with severe FPE: compared to the T0 baseline, patients with T1 lesions showed higher odds (OR = 2.383, *p* = 0.022), and this association was even more pronounced in those with T2 lesions (OR = 4.226, 95% CI: 1.604-11.137, *p* = 0.004).

**Figure 2.**
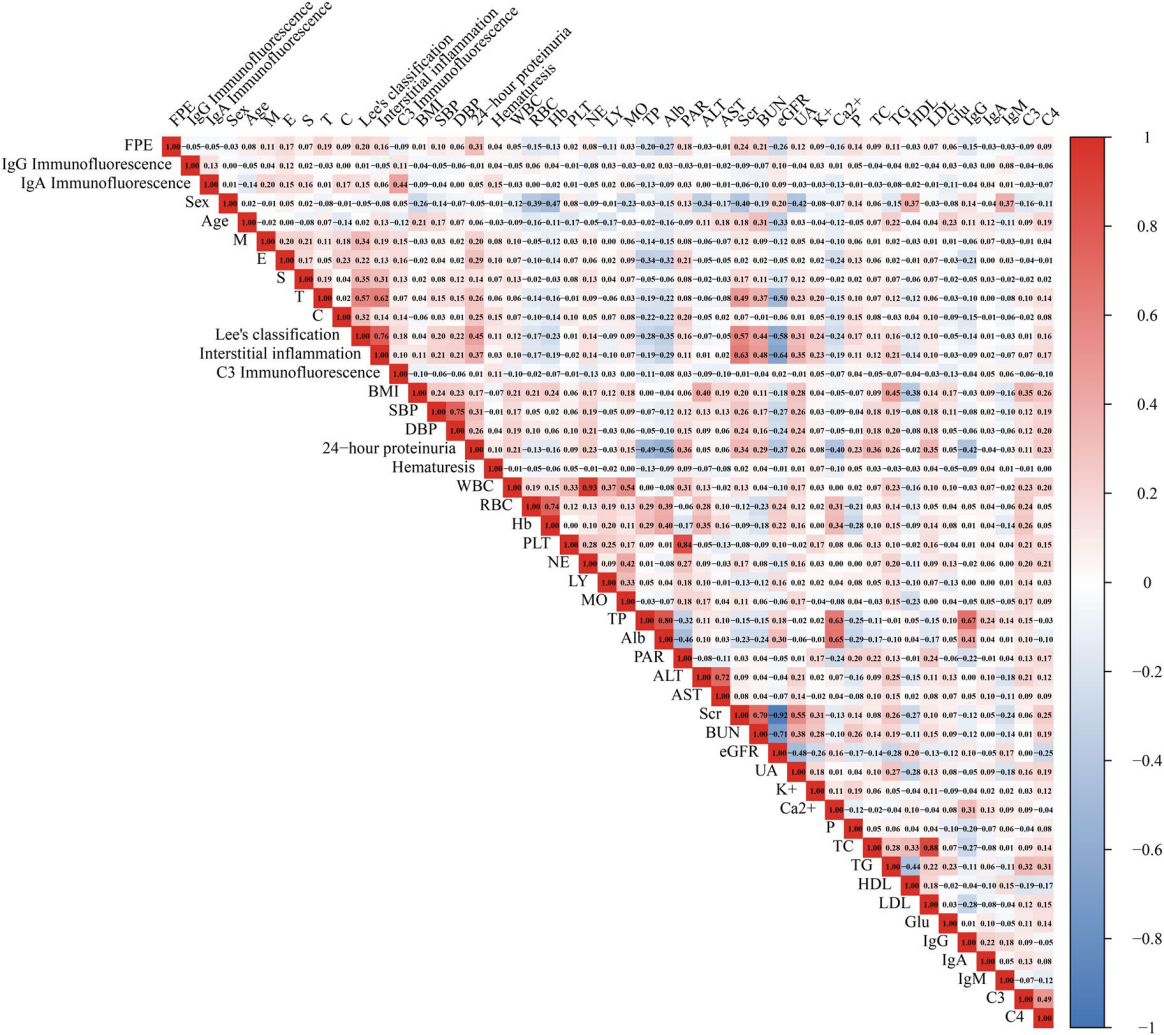
Spearman correlation heatmap of FPE with clinical and pathological characteristics in IgAN.

Spearman correlation analysis (Figure 2 and Supplement Table 2) confirmed positive correlations of FPE with 24-h proteinuria (*r* = 0.31) and Lee’s classification (*r* = 0.20), and a negative correlation with Alb (r = −0.27).

### 3.3. Mediation analysis

In the mediation analysis between FPE severity and hemoglobin levels, adjusted for age, sex, BMI, and systolic blood pressure, distinct patterns emerged. Comparing moderate with mild FPE patients, eGFR showed a statistically significant indirect association [indirect effect = −0.814 (95% CI −1.598, −0.050), *p* = 0.036], whereas the indirect association *via* PAR was not significant (*p* = 0.199). In the severe vs. mild FPE comparison, both eGFR and PAR demonstrated significant indirect associations. The eGFR pathway [indirect effect = −3.074, (95% CI −4.618, −1.649), *p* < 0.001] mediated approximately 30.7% of the total association, while the PAR pathway [indirect effect = −1.492, (95% CI −2.715, −0.283), *p* = 0.013] accounted for 14.9%. Together, these factors explained roughly 45.6% of the statistical association. When comparing severe to moderate FPE, statistical significance was limited to the eGFR pathway [indirect effect = −2.458 (95% CI −4.131, −0.983), *p* < 0.001], which accounted for approximately 27.5% of the total association. In contrast, the PAR pathway was non-significant (*p* = 0.301). Details are shown in Supplementary Table 3.

### 3.4. Short-term renal outcome of different FPE groups after overlap weighting

**Figure 3.**
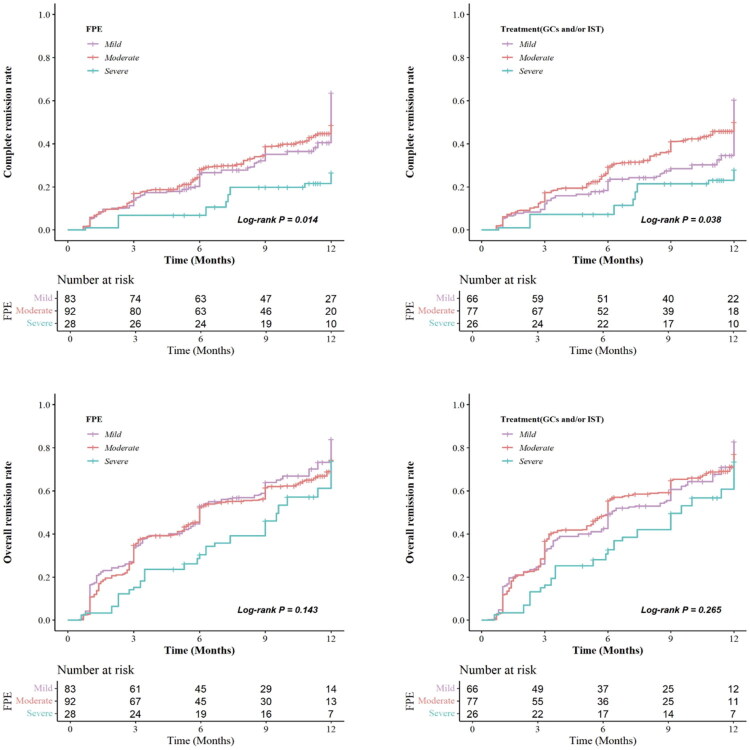
Kaplan–Meier analysis of complete and overall remission rates stratified by foot process effacement severity.

Applying overlap weighting resulted in a sample of 83 patients with mild, 92 with moderate, and 28 with severe FPE in IgAN (Supplement Table 4). Although the SMDs for some covariates did not drop below 0.2, they were still significantly reduced from baseline levels (Supplement Figure 1). Over a median follow-up of 15.4 months, we employed multivariable Cox proportional hazards models to evaluate the impact of FPE on 1-year CR and overall remission, with adjustments for various covariates across models (Supplement Table 5). After adjusting only for age, sex, BMI, eGFR, and baseline proteinuria (Model 1), the severe FPE group exhibited a lower overall remission (HR = 0.659, 95% CI: 0.444-0.978, *p* = 0.038) and CR (HR = 0.426, 95% CI: 0.204-0.891, *p* = 0.023) rates compared to the mild group. After further adjustment for the Oxford MEST-C score (Model 2) and treatment regimen (Model 3), severe FPE remained independently associated with a significantly reduced risk of CR (HR = 0.475, 95% CI: 0.229-0.988, *p* = 0.046). Details are shown in Figure 3. Kaplan-Meier survival analysis also demonstrated a significant difference in CR rates among the three groups over time (log-rank test, *p* = 0.014), and this difference remained significant in the comparison between the groups receiving GCs and/or IST (log-rank test, *p* = 0.038). Details are shown in Figure 3.

## 4. Discussion

This retrospective study involving 1,001 patients with IgAN aimed to investigate the associations between FPE severity and clinical, pathological, and prognostic characteristics. First, we found that greater FPE severity was correlated with higher blood pressure (BP), more severe anemia, impaired renal function, increased proteinuria, and more pronounced systemic inflammation. Second, we observed distinct clinicopathological profiles associated with different levels of FPE severity. Moderate FPE was primarily linked to parameters such as 24-h proteinuria and serum complement levels. In contrast, severe FPE was associated with hyperphosphatemia, persistent Alb depletion, E1 lesions, and progressive tubulointerstitial damage. Furthermore, mediation analysis suggested that the exacerbated anemia observed in patients with severe FPE, compared to those with mild FPE, may be associated with impaired renal function and higher levels of systemic inflammation.

Podocytes play a pivotal role in maintaining the structural integrity and normal function of the glomerular filtration barrier, which is essential for preventing proteinuria [20]. Previous studies have demonstrated that podocyte injury contributes to the development of proteinuria, and podocyte loss predicts its progression [21]. In minimal change disease, the only pathological alteration is diffuse FPE, which indirectly highlights its importance, as this condition is often characterized by massive proteinuria. Furthermore, subtyping of S1 lesions has identified podocyte hypertrophy as the strongest determinant of proteinuria levels [22]. Similarly, a Korean study quantitatively measured FPE length on electron microscopy images and found a significant positive correlation between the degree of FPE and proteinuria [23]. Kim et al. reported that IgAN patients presenting with nephrotic syndrome exhibit more severe FPE and poorer clinical outcomes compared to those without [24]. Our findings are consistent with these observations.

Our study demonstrated that BP increases in tandem with the severity of FPE, suggesting a systemic hemodynamic association. This relationship likely reflects a deleterious feedback loop: IgA1 can stimulate mesangial cells to activate the intrarenal renin-angiotensin system, thereby impairing podocyte adhesion [25]. Conversely, the mechanical strain imposed by chronic hypertension on the glomerular capillary wall may accelerate podocyte thinning and detachment [26]. This reciprocal reinforcement between hemodynamic stress and structural injury potentially drives the progression of podocyte loss and subsequent renal decline.

The integration of ultrastructural FPE severity with light-microscopic findings from the Oxford MEST-C classification reveals critical synergies between different compartments of the nephron. The significantly higher proportions of M1 and E1 in the severe group suggest the intensive mesangial-podocyte cross-talk. Mesangial cells activated by Gd-IgA1 complexes secrete proinflammatory cytokines such as TNF-α and transforming growth factor-β (TGF-β), which directly impair podocyte adhesion and slit diaphragm integrity [27]. Furthermore, the increased prevalence of T2 and C2 lesions among patients with severe FPE demonstrate the global nature of renal injury in these cases. Severe FPE is associated with intense interstitial inflammation, observed in 56.0% of the severe group. This likely results from "proteinuria toxicity," where excessive amounts of Alb and other macromolecules filtered through the damaged podocyte barrier stimulate proximal tubular epithelial cells to produce profibrotic factors, driving tubulointerstitial fibrosis [28].

Beyond local intrarenal inflammation, the role of systemic inflammation in the progression of IgAN has drawn increasing attention. To more comprehensively investigate the relationship between FPE and systemic inflammatory status, we further compared PAR levels across FPE severity levels. PAR, an easily accessible clinical parameter, has been identified as a novel prognostic marker in several inflammatory diseases [29–31] and has also been shown to be an independent risk factor for IgAN progression [32]. Our findings demonstrated that a high PAR level was associated with severe FPE. Moreover, mediation analysis revealed that, in patients with severe FPE, systemic inflammation and impaired renal function may mediate the association between FPE severity and hemoglobin levels.

Multivariate logistic regression analysis identified potential clinical and pathological factors associated with different stages of FPE injury. In the moderate FPE group, higher 24-h proteinuria and serum calcium levels were independent positive correlates, while eGFR and Alb showed negative correlations. Mineral metabolism disorders are a typical feature of chronic kidney disease [33], but the role of serum calcium in the progression of IgA nephropathy remains unclear; a large cohort study of 2,567 IgAN patients found no significant association between serum calcium levels and kidney disease progression events [34]. From a pathological perspective, Lee grades III and IV were associated with moderate FPE, suggesting that once the disease progresses to the stage of overt mesangial proliferation and early sclerosis, podocyte involvement becomes a prominent feature. Similarly, T1 lesions were associated with an increased risk of moderate FPE, further supporting the association between glomerular injury and tubular injury. In the severe FPE group, serum phosphorus level showed a positive correlation. Elevated serum phosphorus has been independently associated with kidney disease progression in IgAN [35], and its association with severe FPE in our study likely reflects worsening renal function. Pathologically, severe FPE was primarily characterized by a higher co‑occurrence rate of E1 lesions, consistent with previous findings [36]. Moreover, T2 lesions also exhibited a correlation; prior studies have linked T2 lesions to poor outcomes and poor response to glucocorticoid therapy [37], which aligns with our short‑term renal prognostic assessment.

Decreased serum complement C3 level is negatively correlated with moderate FPE, while increased C4 level is positively correlated with moderate FPE. Previous studies have found that decreased serum C3 and increased C4 are associated with poor renal prognosis in IgAN [38], which is consistent with the findings of this study. However, no correlation with complement levels was observed in the severe FPE group. Some current studies have linked serum C3 and C4 fractions to disease activity and prognosis [39], and our results suggest that further investigation into the differences of these indicators across different FPE subgroups is necessary.

Severe FPE was identified as an independent predictor of poor short‑term prognosis in IgAN. With a mean follow‑up of 15.4 months, after adjusting for baseline proteinuria, renal function, MEST‑C score, and treatment regimens, patients with severe FPE still showed significantly lower probabilities of achieving CR and overall remission compared with those in the mild group. This finding suggests that ultrastructural damage captures a disease dimension beyond conventional light microscopy and clinical parameters. The observed “prognostic disadvantage” may be attributable to more advanced chronic lesions and irreversible podocyte injury in the severe FPE group. Kaplan‑Meier survival analysis further confirmed that remission rates in the severe FPE group remained consistently low regardless of whether GCs and/or IST were administered. These findings challenge the therapeutic strategy for IgAN patients with severe FPE. Although agents such as cyclosporine can stabilize the podocyte cytoskeleton by modulating RhoA activity [40], previous studies suggest that diffuse podocyte injury may benefit more from early combination immunosuppression [41]. Over the past decade, major advances have been made in targeted therapy for IgAN, driven by a deeper understanding of mucosal immunity, immune‑kidney cell crosstalk, and complement activation [42–44]. Several drugs, including TRF‑budesonide, iptacopan, BAFF/APRIL inhibitors (sibeprenlimab, telitacicept), and endothelin receptor antagonists (sparsentan, atrasentan) have been approved or entered late‑stage clinical trials. In addition, cell‑based therapies have shown promise [45]. However, whether these emerging interventions can effectively improve outcomes in patients with severe FPE remains to be further established.

There are several limitations in this study. First, as this was a multicenter retrospective study of a Chinese cohort with inconsistent IST durations and strategies, there are inherent limitations regarding causal inference and the generalizability of the results to other populations. Prospective, randomized controlled studies are more objective for evaluating the associations between FPE and the clinical features, pathological findings, and prognosis of IgAN. Second, kidney pathology has its own limitations, and the relatively small number of glomeruli in electron microscopy samples may not fully reflect the complete picture of podocyte injury across the entire kidney. Third, only 486 cases (48.6%) were followed up for over one year, and the number of patients with severe FPE was limited. Although overlap weighting was utilized to retain the entire sample and avoid the attrition associated with matching methods, residual imbalance in some covariates persisted after adjustment, which limited the statistical power. Therefore, the results should be interpreted with caution. Finally, due to the relatively short follow-up period of this study, we did not evaluate long-term adverse renal outcomes (such as eGFR decline ≥40%, ESKD, and death).

In summary, our study found that the severity of FPE not only reflects ultrastructural damage but is also closely associated with hemodynamic alterations, systemic inflammatory status, and more severe clinical phenotypes in IgAN. Severe FPE is an independent risk factor for a lower short-term remission rate, and its role in evaluating the prognosis of IgAN warrants further investigation.

## Supplementary Material

Supplementary materials.docx

## Data Availability

The data underlying this article will be shared upon reasonable request to the corresponding author.
