# Supplementary material for: Correlation of foot process effacement with clinicopathological features and short-term prognosis in IgA nephropathy
Source: Ann Med. 2026 May 20;58(1):2674376. doi: 10.1080/07853890.2026.2674376 (PMC13195703; doi:10.1080/07853890.2026.2674376)
Supplement: Supplementary materials.docx [file IANN_A_2674376_SM6052.docx]

| **Supplement Table 1:** Univariate multinomial logistic regression analysis of clinical and pathological indicators associated with foot process effacement | | | | |
| --- | --- | --- | --- | --- |
|  | **Moderate Group** |  | **Severe Group** |  |
| **Variables** | **OR (95% CI)** | ***P*-value** | **OR (95% CI)** | ***P*-value** |
| Sex |  |  |  |  |
| Male | Reference |  | Reference |  |
| Female | 0.972 (0.742-1.275) | 0.838 | 0.735 (0.484-1.115) | 0.147 |
| Age (years) | 1.009 (0.999-1.020) | 0.084 | 1.025 (1.008-1.041) | 0.003 |
| BMI (kg/m^2^) | 0.997 (0.962-1.034) | 0.879 | 1.035 (0.980-1.092) | 0.214 |
| SPB (mmHg) | 1.007 (1.000-1.014) | 0.065 | 1.019 (1.009-1.030) | <0.001 |
| DBP (mmHg) | 1.004 (0.994-1.015) | 0.392 | 1.025 (1.010-1.040) | 0.001 |
| 24h proteinuria (g/24h) | 1.231 (1.133-1.338) | <0.001 | 1.469 (1.334-1.617) | <0.001 |
| WBC (×10^9^/L) | 1.039 (0.973-1.109) | 0.255 | 1.141 (1.039-1.253) | 0.006 |
| RBC (×10^12^/L) | 0.757 (0.628-0.912) | 0.003 | 0.438 (0.324-0.593) | <0.001 |
| Hb (g/L) | 0.993 (0.987-1.000) | 0.044 | 0.976 (0.966-0.986) | <0.001 |
| Plt (×10^9^/L) | 1.000 (0.998-1.002) | 0.746 | 1.001 (0.999-1.004) | 0.343 |
| eGFR (ml/min/1.73m^2^) | 0.990 (0.986-0.994) | <0.001 | 0.972 (0.965-0.980) | <0.001 |
| UA (umol/L) | 1.002 (1.000-1.003) | 0.013 | 1.003 (1.001-1.005) | <0.001 |
| Alb (g/L) | 0.945 (0.921-0.970) | <0.001 | 0.820 (0.791-0.850) | <0.001 |
| ALT (U/L) | 1.005 (0.998-1.012) | 0.177 | 0.985 (0.969-1.003) | 0.095 |
| AST (U/L) | 1.010 (0.998-1.022) | 0.113 | 0.996 (0.974-1.018) | 0.704 |
| PAR | 1.079 (1.013-1.150) | 0.018 | 1.351 (1.247-1.464) | <0.001 |
| TG (mmol/L) | 1.126 (1.013-1.252) | 0.029 | 1.240 (1.078-1.428) | 0.003 |
| Serum HDL (mmol/L) | 0.957 (0.676-1.354) | 0.803 | 0.808 (0.463-1.409) | 0.452 |
| Serum LDL (mmol/L) | 1.035 (0.910-1.178) | 0.597 | 1.464 (1.240-1.728) | <0.001 |
| Glu (mmol/L) | 1.061 (0.933-1.207) | 0.366 | 1.128 (0.935-1.360) | 0.208 |
| Serum potassium (mmol/L) | 1.302 (0.925-1.831) | 0.130 | 2.521 (1.534-4.143) | <0.001 |
| Serum calcium (mmol/L) | 0.797 (0.298-2.134) | 0.651 | 0.003 (0.001-0.011) | <0.001 |
| Phosphorus (mmol/L) | 1.443 (0.731-2.849) | 0.290 | 13.932 (5.776-33.608) | <0.001 |
| IgG (g/L) | 0.978 (0.937-1.022) | 0.327 | 0.805 (0.750-0.865) | <0.001 |
| lgA (g/L) | 1.031 (0.933-1.140) | 0.548 | 0.907 (0.755-1.089) | 0.294 |
| IgM (g/L) | 1.017 (0.815-1.270) | 0.880 | 0.823 (0.563-1.204) | 0.316 |
| C3 (g/L) | 0.326 (0.171-0.621) | <0.001 | 1.162 (0.557-2.423) | 0.689 |
| C4 (g/L) | 3.509 (0.575-21.420) | 0.174 | 27.821 (2.369-326.674) | 0.008 |
| Oxford histologic score |  |  |  |  |
| M0 | Reference |  | Reference |  |
| M1 | 2.327 (1.302-4.160) | 0.004 | 5.284 (1.248-22.363) | 0.024 |
| E0 | Reference |  | Reference |  |
| E1 | 1.500 (1.136-1.981) | 0.004 | 3.333 (2.168-5.123) | <0.001 |
| S0 | Reference |  | Reference |  |
| S1, S2 | 1.471 (1.117-1.938) | 0.006 | 1.305 (0.849-2.008) | 0.225 |
| T0 | Reference |  | Reference |  |
| T1 | 1.558 (1.057-2.297) | 0.025 | 2.657 (1.517-4.654) | <0.001 |
| T2 | 1.706 (0.840-3.465) | 0.139 | 11.458 (5.521-23.780) | <0.001 |
| C0 | Reference |  | Reference |  |
| C1 | 1.224 (0.931-1.608) | 0.148 | 1.470 (0.946-2.285) | 0.087 |
| C2 | 1.258 (0.607-2.604) | 0.537 | 5.123 (2.294-11.442) | <0.001 |
| Lee's classification |  |  |  |  |
| I-II | Reference |  | Reference |  |
| III | 4.448 (2.809-7.043) | <0.001 | 1.452 (0.679-3.104) | 0.337 |
| IV | 3.690 (2.240-6.080) | <0.001 | 2.250 (1.019-4.970) | 0.045 |
| V | 3.480 (1.964-6.165) | <0.001 | 9.000 (4.165-19.449) | <0.001 |
| Interstitial inflammation |  |  |  |  |
| Mild | Reference |  | Reference |  |
| Moderate | 1.582 (1.152-2.174) | 0.005 | 1.578 (0.862-2.889) | 0.139 |
| Severe | 1.501 (1.071-2.104) | 0.019 | 4.282 (2.467-7.431) | <0.001 |
| C3 Immunofluorescence |  |  |  |  |
| Negative | Reference |  | Reference |  |
| Weakly positive | 1.243 (0.785-1.967) | 0.353 | 1.297 (0.659-2.554) | 0.452 |
| Strongly positive | 0.804 (0.537-1.203) | 0.289 | 0.698 (0.377-1.294) | 0.254 |
| IgG Immunofluorescence |  |  |  |  |
| Negative | Reference |  | Reference |  |
| Positive | 0.644 (0.418-0.991) | 0.045 | 0.782 (0.401-1.525) | 0.471 |
| IgA Immunofluorescence |  |  |  |  |
| Weakly positive | Reference |  | Reference |  |
| Strongly positive | 0.713 (0.517-0.984) | 0.040 | 0.884 (0.531-1.470) | 0.634 |
| **Note:** The reference group for the dependent variable (foot process effacement) is the mild group. OR, adjusted odds ratio; CI, confidence interval. | | | | |

**Supplement Table 2:** Spearman correlation analysis between various risk factors and FPE

| **Correlation** | **Variable** | **Correlation coefficient** |
| --- | --- | --- |
| **Moderately correlated** | 24-hour proteinuria | 0.31 |
|  | Alb | -0.27 |
|  | eGFR | -0.26 |
|  | Scr | 0.24 |
|  | BUN | 0.21 |
|  | Lee's classification | 0.20 |
| **Weak correlation** | T | 0.19 |
|  | PAR | 0.18 |
|  | E | 0.17 |
|  | Interstitial inflammation | 0.16 |
|  | Ca^2+^ | -0.16 |
|  | IgG | -0.15 |
| **Extremely weak correlation** | IgA | -0.03 |
|  | IgM | -0.03 |
|  | MONO | 0.03 |
|  | IgG Immunofluorescence | -0.05 |
|  | IgA Immunofluorescence | -0.05 |

**Supplement Table 3:** Association of FPE severity with hemoglobin: mediation analysis involving eGFR and PAR

| **Comparison Group** | **Mediator** | **Indirect effect** | **95% CI** | ***P*** | **Direct effect** | **Total effect** |
| --- | --- | --- | --- | --- | --- | --- |
| Moderate vs Mild | eGFR | -0.814 | [-1.598, -0.050] | 0.036 | 0.928 | 0.114 |
| Moderate vs Mild | PAR | 0.203 | [-0.117, 0.550] | 0.199 | -0.090 | 0.114 |
| Severe vs Mild | eGFR | -3.074 | [-4.618, -1.649] | <0.001 | -6.945 | -10.019 |
| Severe vs Mild | PAR | -1.492 | [-2.715, -0.283] | 0.013 | -8.527 | -10.019 |
| Severe vs Moderate | eGFR | -2.458 | [-4.131, -0.983] | <0.001 | -6.480 | -8.938 |
| Severe vs Moderate | PAR | -0.730 | [-2.061, 0.684] | 0.301 | -8.208 | -8.938 |

All models adjusted for age, sex, BMI, and systolic blood pressure

**Supplement Table 4:** Baseline characteristics before and after overlap weighting of followed up patients

|  | | **Before overlap weighting** | | | | **After overlap weighting** | | | |
| --- | --- | --- | --- | --- | --- | --- | --- | --- | --- |
| **Characteristics** | | **Mild Group (n=187)** | **Moderate Group (n=256)** | **Severe Group (n=43)** | **Maximum Pairwise SMD** | **Mild Group (n=83)** | **Moderate Group (n=92)** | **Severe Group (n=28)** | **Maximum Pairwise SMD** |
| Age (years) | | 36.83 (10.99) | 38.50 (12.00) | 41.12 (15.53) | 0.217 | 37.08 (10.96) | 37.28 (11.60) | 39.91 (14.86) | 0.144 |
| SBP (mmHg) | | 129.46 (17.43) | 131.61 (18.66) | 137.65 (21.54) | 0.279 | 130.76 (17.66) | 131.21 (18.75) | 135.39 (19.95) | 0.162 |
| RBC (×10^12^/L) | | 4.65 (0.69) | 4.47 (0.72) | 4.36 (0.60) | 0.283 | 4.57 (0.71) | 4.55 (0.68) | 4.39 (0.61) | 0.183 |
| Hb (g/L) | | 133.37 (19.80) | 128.63 (22.43) | 125.56 (19.83) | 0.255 | 130.45 (20.35) | 130.17 (21.71) | 126.50 (20.30) | 0.128 |
| NEUT (×10^9^/L) | | 4.30 (1.70) | 4.60 (1.90) | 5.12 (2.48) | 0.262 | 4.54 (1.85) | 4.59 (2.09) | 4.82 (2.12) | 0.092 |
| LYMPH (×10^9^/L) | | 1.86 (0.57) | 1.78 (0.54) | 1.62 (0.52) | 0.295 | 1.85 (0.59) | 1.82 (0.55) | 1.66 (0.48) | 0.241 |
| 24h-UTP (mg/24h) | | 1500.05 (1533.63) | 2308.29 (2141.34) | 3381.61 (1989.00) | 0.671 | 1817.59 (1920.55) | 2073.17 (2010.96) | 2987.21 (1900.48) | 0.403 |
| TP (g/L) | | 68.34 (7.69) | 67.45 (8.04) | 59.75 (11.34) | 0.594 | 67.64 (8.53) | 67.11 (8.15) | 62.35 (10.61) | 0.372 |
| Alb (g/L) | | 41.15 (5.01) | 39.14 (5.38) | 33.74 (8.59) | 0.731 | 40.01 (5.55) | 39.52 (5.30) | 36.07 (7.94) | 0.393 |
| Scr (μmol/L) | | 94.18 (39.59) | 112.90 (60.74) | 127.39 (63.79) | 0.408 | 100.99 (42.94) | 106.42 (56.86) | 128.40 (66.43) | 0.318 |
| BUN (mmol/L) | | 5.48 (2.05) | 6.09 (2.38) | 6.73 (2.64) | 0.355 | 5.75 (2.27) | 5.98 (2.32) | 6.78 (2.72) | 0.275 |
| eGFR (ml/min/1.73 m^2^) | | 86.54 (30.30) | 72.54 (31.27) | 67.43 (35.68) | 0.395 | 79.71 (29.74) | 77.72 (32.07) | 67.02 (34.14) | 0.261 |
| UA (umol/L) | | 374.42 (111.82) | 405.56 (103.75) | 383.75 (91.90) | 0.201 | 388.89 (110.52) | 390.08 (100.71) | 384.80 (92.55) | 0.035 |
| K^+^ (mmol/L) | | 4.00 (0.37) | 4.06 (0.40) | 4.10 (0.56) | 0.150 | 4.04 (0.38) | 4.06 (0.40) | 4.14 (0.56) | 0.140 |
| Ca^2+^ (mmol/L) | | 2.30 (0.13) | 2.29 (0.14) | 2.19 (0.18) | 0.453 | 2.29 (0.14) | 2.29 (0.13) | 2.23 (0.18) | 0.270 |
| P (mmo/L) | | 1.09 (0.18) | 1.12 (0.20) | 1.18 (0.18) | 0.332 | 1.10 (0.18) | 1.11 (0.19) | 1.18 (0.17) | 0.275 |
| TG (mmol/L) | | 1.84 (1.19) | 2.14 (1.48) | 2.14 (1.46) | 0.152 | 1.93 (1.22) | 2.00 (1.39) | 2.20 (1.58) | 0.126 |
| TC (mmol/L) | | 5.12 (1.32) | 5.22 (1.42) | 5.51 (1.77) | 0.168 | 5.19 (1.33) | 5.22 (1.41) | 5.32 (1.65) | 0.058 |
| LDL (mmol/L) | | 3.18 (1.00) | 3.19 (1.08) | 3.55 (1.66) | 0.181 | 3.20 (1.01) | 3.23 (1.07) | 3.36 (1.54) | 0.086 |
| IgG (g/L) | | 11.71 (2.73) | 11.61 (3.13) | 9.97 (3.37) | 0.367 | 11.59 (2.82) | 11.50 (3.10) | 10.48 (3.33) | 0.236 |
| lgA (g/L) | | 3.43 (1.73) | 3.47 (1.31) | 3.20 (1.35) | 0.129 | 3.41 (1.38) | 3.37 (1.28) | 3.16 (1.28) | 0.128 |
| IgM (g/L) | | 1.25 (0.51) | 1.35 (0.66) | 1.15 (0.33) | 0.256 | 1.29 (0.51) | 1.28 (0.52) | 1.15 (0.33) | 0.219 |
| C3 (g/L) | | 1.05 (0.20) | 0.97 (0.18) | 1.04 (0.23) | 0.252 | 1.01 (0.20) | 1.01 (0.21) | 1.02 (0.23) | 0.052 |
| C4 (g/L) | | 0.25 (0.07) | 0.26 (0.08) | 0.28 (0.09) | 0.225 | 0.26 (0.08) | 0.26 (0.08) | 0.28 (0.09) | 0.170 |
| PAR | | 6.55 (1.87) | 6.90 (2.16) | 9.03 (3.68) | 0.577 | 6.87 (2.01) | 7.05 (2.21) | 7.99 (2.97) | 0.296 |
| Lee's classification | I | 1 (0.5) | 0 (0.0) | 0 (0.0) | 0.714 | 0.0 (0.0) | 0.0 (0.0) | 0.0 (0.0) | 0.467 |
|  | II | 37 (19.8) | 15 (5.9) | 1 (2.3) |  | 8.8 (10.6) | 9.0 (9.8) | 0.6 (2.0) |  |
|  | III | 88 (47.1) | 146 (57.0) | 12 (27.9) |  | 41.7 (50.2) | 44.0 (47.9) | 7.8 (28.3) |  |
|  | IV | 42 (22.5) | 68 (26.6) | 15 (34.9) |  | 22.3 (26.8) | 27.6 (30.0) | 11.3 (41.0) |  |
|  | V | 19 (10.2) | 27 (10.5) | 15 (34.9) |  | 10.3 (12.4) | 11.4 (12.4) | 7.9 (28.7) |  |
| M | 0 | 15 (8.0) | 10 (3.9) | 1 (2.3) | 0.175 | 4.2 (5.0) | 4.5 (4.9) | 0.6 (2.0) | 0.109 |
|  | 1 | 172 (92.0) | 246 (96.1) | 42 (97.7) |  | 79.0 (95.0) | 87.5 (95.1) | 27.0 (98.0) |  |
| E | 0 | 116 (62.0) | 147 (57.4) | 17 (39.5) | 0.307 | 49.1 (59.0) | 53.3 (57.9) | 12.2 (44.1) | 0.201 |
|  | 1 | 71 (38.0) | 109 (42.6) | 26 (60.5) |  | 34.1 (41.0) | 38.7 (42.1) | 15.4 (55.9) |  |
| S | 0 | 70 (37.4) | 80 (31.2) | 12 (27.9) | 0.159 | 25.8 (31.0) | 29.3 (31.8) | 6.9 (24.9) | 0.102 |
|  | 1 | 117 (62.6) | 175 (68.4) | 31 (72.1) |  | 57.4 (69.0) | 62.7 (68.2) | 20.7 (75.1) |  |
|  | 2 | 0 (0.0) | 1 (0.4) | 0 (0.0) |  | 0.0 (0.0) | 0.0 (0.0) | 0.0 (0.0) |  |
| T | 0 | 142 (75.9) | 181 (70.7) | 20 (46.5) | 0.439 | 58.5 (70.3) | 63.0 (68.5) | 12.8 (46.5) | 0.342 |
|  | 1 | 36 (19.3) | 57 (22.3) | 15 (34.9) |  | 19.5 (23.4) | 22.5 (24.5) | 10.4 (37.7) |  |
|  | 2 | 9 (4.8) | 18 (7.0) | 8 (18.6) |  | 5.2 (6.2) | 6.4 (7.0) | 4.4 (15.8) |  |
| C | 0 | 106 (56.7) | 125 (48.8) | 16 (37.2) | 0.324 | 43.0 (51.7) | 46.5 (50.6) | 11.1 (40.2) | 0.179 |
|  | 1 | 76 (40.6) | 120 (46.9) | 22 (51.2) |  | 37.2 (44.8) | 41.2 (44.8) | 14.5 (52.5) |  |
|  | 2 | 5 (2.7) | 11 (4.3) | 5 (11.6) |  | 3.0 (3.5) | 4.2 (4.6) | 2.0 (7.4) |  |
| Interstitial inflammation | Mild | 76 (40.6) | 68 (26.6) | 7 (16.3) | 0.548 | 25.9 (31.2) | 26.4 (28.7) | 4.4 (16.0) | 0.373 |
|  | Moderate | 65 (34.8) | 110 (43.0) | 11 (25.6) |  | 31.6 (38.0) | 35.1 (38.1) | 7.5 (27.2) |  |
|  | Severe | 46 (24.6) | 78 (30.5) | 25 (58.1) |  | 25.6 (30.8) | 30.5 (33.2) | 15.7 (56.8) |  |
| C3 Immunofluorescence | Negative | 22 (11.8) | 41 (16.0) | 4 (9.3) | 0.364 | 10.0 (12.1) | 11.2 (12.1) | 1.8 (6.4) | 0.272 |
|  | Weakly positive | 30 (16.0) | 70 (27.3) | 16 (37.2) |  | 16.5 (19.8) | 22.0 (23.9) | 10.1 (36.6) |  |
|  | Strongly positive | 135 (72.2) | 145 (56.6) | 23 (53.5) |  | 56.7 (68.1) | 58.8 (64.0) | 15.7 (57.0) |  |

**Abbreviations:** SMD, standardized mean difference.

| **Supplement table 5:** Multivariable Cox proportional hazards model for overall and complete remission by FPE status in IgAN patients after overlap weighting. | | | | | | | | | |
| --- | --- | --- | --- | --- | --- | --- | --- | --- | --- |
| **Variable** | **Model 1** | | | **Model 2** | | | **Model 3** | | |
|  | **HR** | **95% Cl** | ***P*** | **HR** | **95% Cl** | ***P*** | **HR** | **95% Cl** | ***P*** |
| **OR** | | | | | | | | | |
| Mild | Reference | | | | | | | | |
| Moderate | 0.872 | 0.675-1.127 | 0.294 | 0.857 | 0.662-1.109 | 0.240 | 0.853 | 0.658-1.105 | 0.229 |
| Severe | 0.659 | 0.444-0.978 | 0.038 | 0.697 | 0.467-1.040 | 0.077 | 0.685 | 0.458-1.026 | 0.067 |
| **CR** | | | | | | | | | |
| Mild | Reference | | | | | | | | |
| Moderate | 0.965 | 0.710-1.312 | 0.822 | 0.967 | 0.713-1.311 | 0.827 | 0.978 | 0.719-1.329 | 0.887 |
| Severe | 0.426 | 0.204-0.891 | 0.023 | 0.464 | 0.224-0.963 | 0.039 | 0.475 | 0.229-0.988 | 0.046 |
| Model 1: Adjusted for age, sex, BMI, eGFR, and baseline proteinuria. Model 2: Model 1 and MEST-C scores by the Oxford classification. Model 3: Model 2 and treatment. | | | | | | | | | |

**Supplement Figure 1:** Love plot revealing the standardization mean differences before and after overlap weighting.


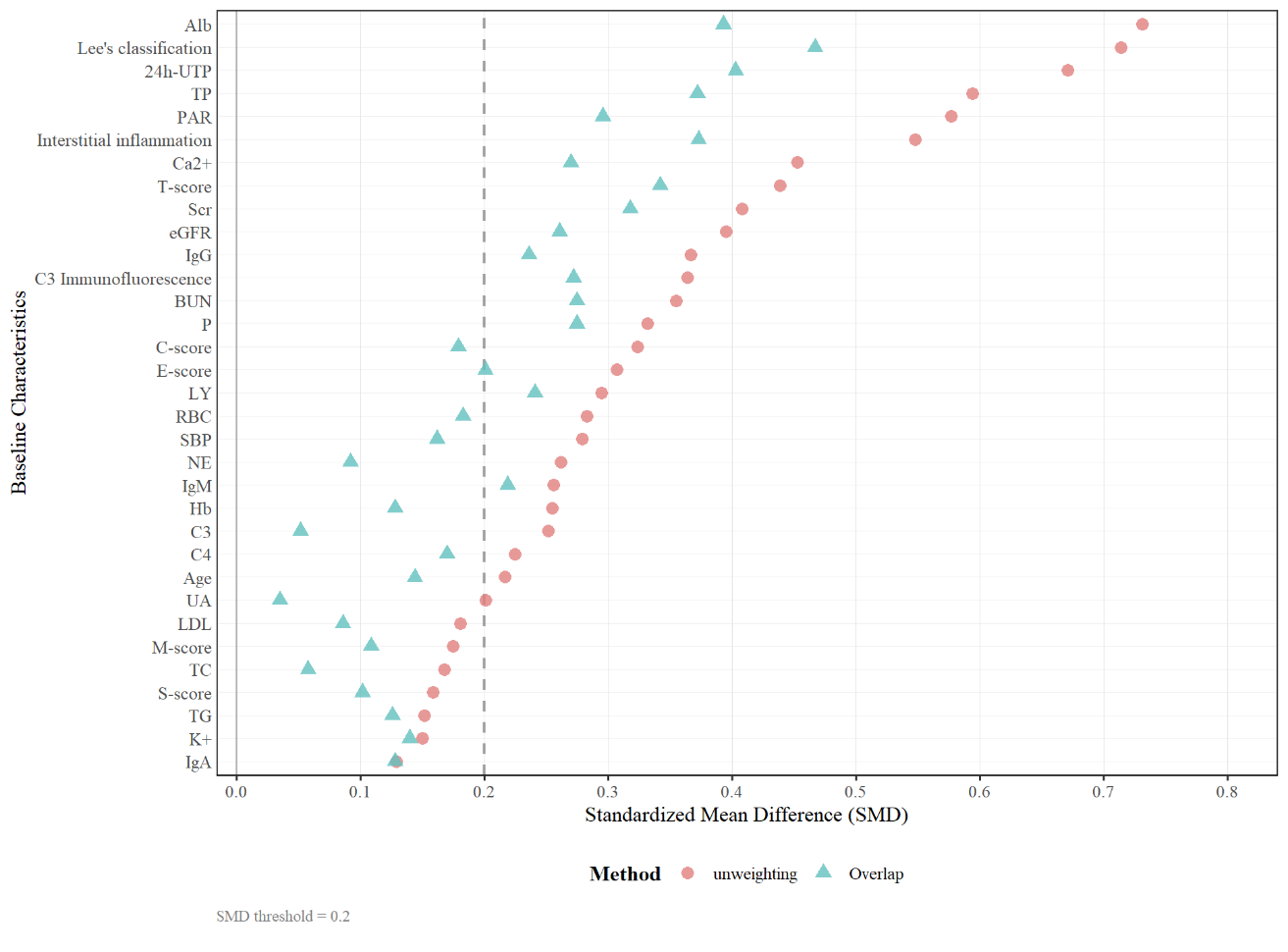


**Figure legend:** Love plot illustrating the SMDs for key baseline variables before and after weighting. Each row on the y-axis represents a covariate; the x-axis shows the absolute value of the SMD. Red circles denote unweighted data, blue triangles show SMDs after overlap weighting. The vertical dashed line at 0.2 indicates a commonly used threshold below which covariate balance is considered satisfactory.
